# Control of occlusion of middle cerebral artery in perinatal and neonatal mice with magnetic force

**DOI:** 10.1186/s13041-018-0389-0

**Published:** 2018-08-29

**Authors:** Jie-Min Jia, Chuanqi Peng, Yihui Wang, Jie Zheng, Woo-Ping Ge

**Affiliations:** 1 Children’s Research Institute, University of Texas Southwestern Medical Center, Dallas, TX USA; 2 School of Life Sciences, Westlake University, Hangzhou, China; 3 Department of Chemistry and Biochemistry, University of Texas at Dallas, Richardson, TX USA; 4 Department of Neurology, Tongji Hospital, Tongji Medical College, Huazhong University of Science and Technology, Wuhan, China; 5 Department of Pediatrics, University of Texas Southwestern Medical Center, Dallas, TX USA; 6 Departments of Neuroscience, University of Texas Southwestern Medical Center, Dallas, TX USA; 7 Department of Neurology and Neurtherapeutics, University of Texas Southwestern Medical Center, Dallas, TX USA; 8 Harold C. Simmons Comprehensive Cancer Center, University of Texas Southwestern Medical Center, Dallas, TX USA

**Keywords:** Magnetic force, Magnetic nanoparticles, Ischemic perinatal stroke, Ischemic neonatal stroke, SIMPLE, Distal MCA

## Abstract

Ischemic perinatal stroke (IPS) is common, resulting in significant mortality and morbidity. In such cases, the incidence of unilateral arterial cerebral infarction is often occluded in the middle cerebral artery (MCA), leading to focal ischemia. In adult rodents, blockage of MCA is the most frequently used strategy for ischemic stroke study. However, modeling MCA occlusion (MCAo) in postnatal day 0–7 (P0–7) mouse pups for IPS study has not been accomplished. Here we occluded the dMCA by inducing the accumulation of magnetic particles (MPs) administered through the superficial temporal vein of mice between P0 and P7, which we called neonatal or perinatal SIMPLE (Stroke Induced with Magnetic Particles). SIMPLE produced either permanent or transient occlusion in the dMCA of perinatal and neonatal mice. Permanent MCA occlusion with SIMPLE resulted in cerebral infarction and neuronal death in the brain. SIMPLE can also be used to reliably produce focal ischemic stroke in neonatal or perinatal mouse brains. As a result, SIMPLE allows the modeling of IPS or focal ischemic stroke for further mechanistic studies in mice, with particular utility for mimicking transient focal ischemia in human pre-term babies, which for the first time here has been accomplished in mice.

**Electronic supplementary material:**

The online version of this article (10.1186/s13041-018-0389-0) contains supplementary material, which is available to authorized users.

## Introduction

**Scheme 1:**
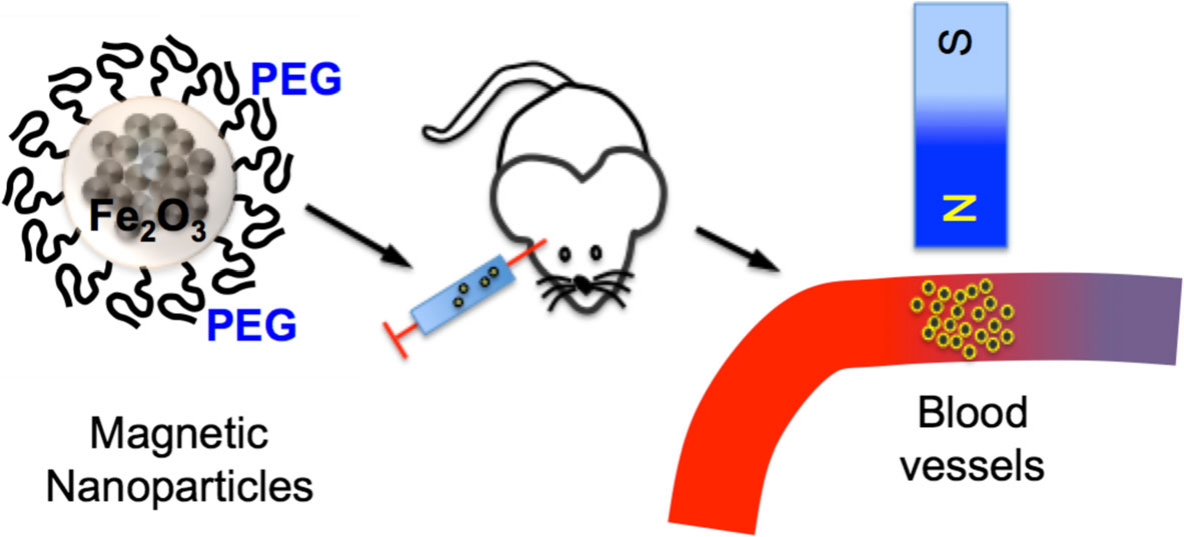
Neonatal or perinatal SIMPLE. We occluded the dMCA or focal microcirculation by inducing the accumulation of magnetic particles (MPs) administered through the superficial temporal vein of mice, which we called neonatal or perinatal SIMPLE (Stroke Induced with Magnetic ParticLEs)

Ischemic perinatal stroke (IPS) in humans is defined as cerebral ischemia that occurs from 20 weeks of gestation through the 28th postnatal day [1]. The incidence of stroke in this age group ranks second only to the incidence in elderly persons, ranging between 1: 1600 and 1: 5000 [1–3]. IPS can result in cerebral palsy, epilepsy, language development delay and other complications [1, 2, 4]. The incidence of unilateral arterial cerebral infarction (ACI) in newborn infants is approximately 1:2300, and occurs most often in the middle cerebral artery (MCA) [2]. The brains of mice on postnatal days (P)0–5 correspond to similar developmental stages to the brains of human pre-term fetuses at of gestation (< 32 weeks) [5, 6]. Because of their small size and low body weight, the common carotid arteries and MCA in the mouse pups are small in diameter and fragile compared to those of adult mice, creating a surgical challenge that has proved insurmountable thus far. Thus, the youngest age in mice at which the MCA can be reversibly occluded by sutures is P9 [5]. Although conventional MCA occlusion (MCAo) can be performed in P7 rats [7–10], unfortunately MCAo is not feasible for mouse pups this young. However, it is highly desirable to model IPS in mice because they are the most commonly genetically edited species. Other approaches have therefore been developed for use in mouse pups, including common carotid ligation followed by a hypoxic episode [11–13], ibotenate injection [14] and dye-mediated photothrombosis (e.g. using Rose-Bengal or erythrosine B) [15–17], but these procedures lead to global hypoxia or permanent focal occlusion and do not allow precise control of reperfusion in blood vessels, and some require overly invasive surgery. Thus, it remains challenging to produce transient focal ischemia in perinatal and neonatal mice to model IPS and study the resulting disruption of the neuron-glia-vasculature network. This study aims to implement and optimize SIMPLE technology [18] to model IPS in perinatal and neonatal mice, thereby providing a convenient strategy to produce focal ischemia in mice of such young ages (see Scheme 1). We use magnetic nanoparticles to occlude the distal MCA (dMCA) of pups as early as P0. SIMPLE does not involve any arterial surgery in the brain, which obviates the difficulty of performing delicate surgery on pups [6] and enables perinatal and neonatal stroke study in a manner that is convenient, potentially expand the scale of investigating IPS. Combined with genetically engineered mouse pups, it provides deep mechanistic insight into the pathology of IPS.

## Results

### Characterizations of magnetic particles

**Fig. 1:**
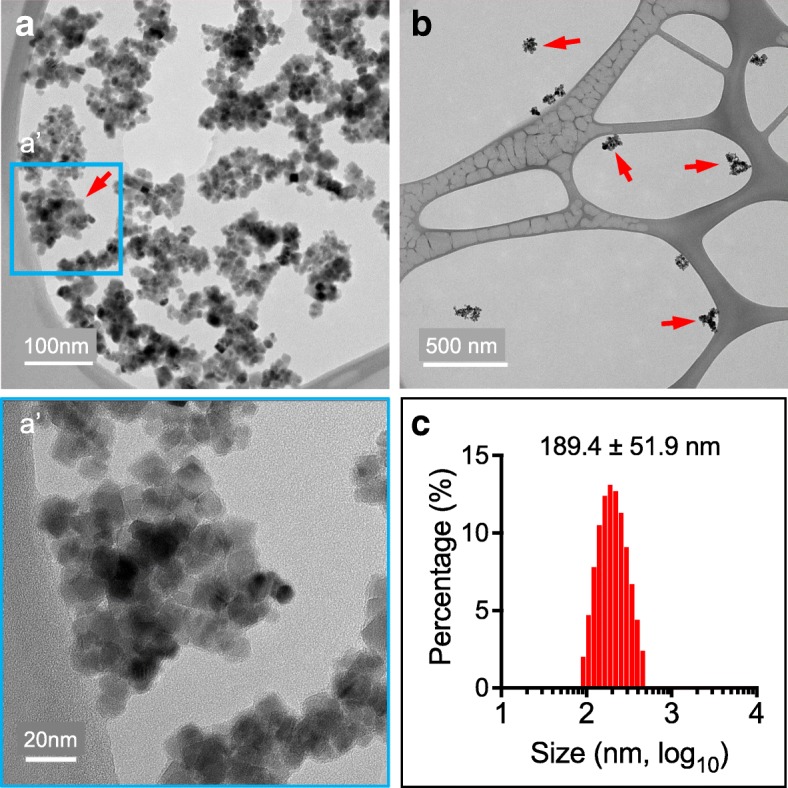
Characterization of the magnetic nanoparticles that we used for blood occlusion. The transmission electron microscopy images were taken by a transmission electron microscope. **a**, **b** Images of MPs with transmission electrical microscopy. 1 mg/ml (**a**) and 0.1 mg/ml (**b**), (**a’**) inset from (**a**). **c**. The diameter of MPs (nanoclusters) was obtained with a particle size analyzer

A critical step for the development of this approach is the design of the magnetic particles. The size, the core materials, and the coating materials of the particles are critical for their toxicity, and circulation time in the bloodstream. In water these particles behave as spheres stabilized by strong H-bridge interactions between dextran and water. Based on our TEM imaging, these particles consist of 10–20 nm sized magnetite crystals and their aggregates embedded into a dextran matrix (Fig. 1). After testing multiple magnetic nanoparticles with sizes ranging from 50- to 200-nm, we found PEG-2000 coated nanoparticles (189.4 ± 51.9 nm, Fig. 1) was most suitable for our following occlusion in neonatal and perinatal brains.

### Focal MP occlusion in perinatal and neonatal brains

**Fig. 2:**
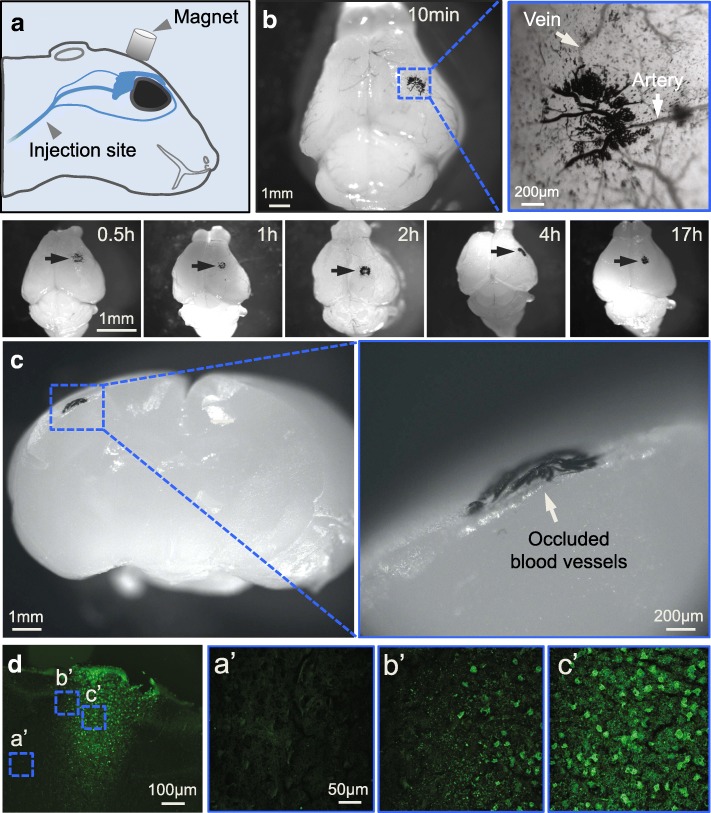
Producing focal occlusion in perinatal and neonatal mouse brains with SIMPLE. **a**. Schematic illustration of the location of the micro-magnet and the site of MP injection. **b**. MP aggregation (arrows) in blood vessels of multiple P3 mice at different time points (10 min, 0.5 h, 1 h, 2 h, 4 h and 17 h; MP dose, 100 μg/g). Right panel, higher magnification image of MP occlusion in a P3 mouse brain after 10 min SIMPLE. **c**. MP aggregation after 10 min SIMPLE in a P7 mouse brain. Inset, high-magnification image of pial vessels occluded with MPs (white arrow). **d**. FJC staining of a brain section from a P3 mouse that was subjected to 24 h SIMPLE. (**a’**–**c’**) Insets from (**d**), indicating regions of non-infarct control region (**a’**), peri-infarct region (**b’**), and infarct region (**c’**)

To produce a focal occlusion in the perinatal brain, MPs (see dose information in Table 1) were administered through the superficial temporal vein of P0–7 mice (Fig. 2a). A cylindrical micro-magnet of 1 mm diameter was glued onto the intact skull. MP occlusion was reliably formed at 10 min, 30 min, 1 h, 2 h, 4 h and 17 h post-SIMPLE in perinatal and neonatal mice (Fig. 2b). Black MP aggregation could be observed in microvessels (Fig. 2b, c). We found most of the MP occlusion located in the pial blood vessels, rather than in their downstream penetrating vessels (Fig. 2c). At 24 h post-SIMPLE, we detected notable neuronal degeneration in multiple layers of the cerebral cortex using Fluoro-Jade C staining (FJC, Fig. 2d). In contrast to neighboring regions, there were many FJC-positive neurons in the occluded region (Fig. 2d). These results suggested that the penetrating blood vessels became dysfunctional after their upstream vessels at the pia were blocked with MPs, which resulted in neuronal degeneration in the deeper layer of the cerebral cortex. In addition, these results indicated that SIMPLE is able to introduce a focal ischemic brain injury in perinatal (P0–3) and neonatal (P4–9) mice.

**Table 1:** Doses of injected MP for modeling IPS

Pup body weight	Doses for permanent occlusion (μg/g b.w.)	Doses for transient occlusion (μg/g b.w.)
2 g	20–100	10–15
3 g	30–100	15–21
4 g	40–100	20–28
5 g	50–100	25–30
6 g	60–100	30–36
7 g	70–100	35–42

Differential injection doses of MPs for modeling permanent or transient occlusion by SIMPLE technology based on mouse pup body weight

**Fig. 3:**
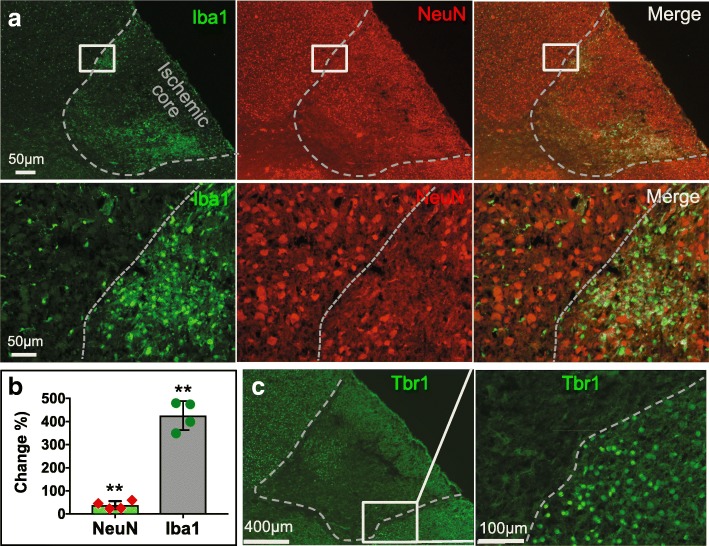
Neurons and microglia in the brain with ischemia produced by SIMPLE. **a**. Neurons (red, anti-NeuN) and microglia (green, anti-Iba-1) in sections from a P7 mouse brain that underwent permanent occlusion with MPs for 3 d. Higher magnification images are shown the lower panel. **b**. Quantification of changes of neuronal and microglial cell density. We compared the ischemic region to the peri-infarct region. **, *p* < 0.005 **c**. Staining for neurons with anti-Tbr1

To further examine the microglial or macrophage aggregation and neuronal loss in the ischemic perinatal brains, we stained the brain sections with microglia/macrophage marker anti-Iba1 and neuron makers anti-NeuN and anti-Tbr1 (Fig. 3). We observed that Iba1^+^ cells accumulated in the right ischemic core region at cell density 4 times higher (426.3 ± 38.4%, *n* = 4) than that of the corresponding region of the contralateral hemisphere. In contrast, the density of neurons labeled with anti-NeuN in the same area decreased by over 60% relative to the corresponding contralateral region (36.0 ± 10.2%, *n* = 4, Fig. 3a, b). We also obtained a similar result after we labeled neurons with anti-Tbr1 (Fig. 3c). Both stainings indicated substantial neuronal loss in the ischemic areas. Taken together, these results demonstrate that SIMPLE causes neuronal degeneration and microglial and/or macrophage aggregation in the ischemic regions of the mouse pup brains.

### Focal ischemia by occluding the dMCA

**Fig. 4:**
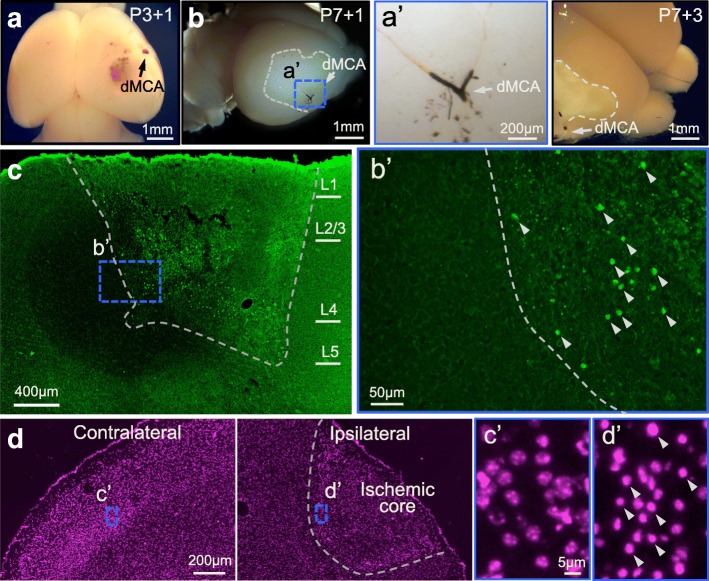
Producing focal dMCA occlusion in perinatal and neonatal brains by SIMPLE. **a**. Permanent MP occlusion (arrowhead) was formed in the dMCA of a P3 mouse (~ 2 g) after 20 min SIMPLE (MP dose of 50 μg/g). Magnet was removed after 20 min, which is long enough to form permanent MP occlusion. The pup was sacrificed at 24 h after SIMPLE. **b**. Permanent MP occlusion was formed in the dMCA of a P7 mouse (~ 4 g) after 20 min SIMPLE (MP dose of 50 μg/g). The pup was sacrificed 17 h later (left panel). The infarct area is highlighted with a dashed line. (**a’**) High-magnification inset from (b) showing MP aggregation in the dMCA (middle panel). Severe brain infarction in the right hemisphere at 3 d after 20 min SIMPLE in a P7 mouse (right panel). MP aggregation was still observed 3 d after 20 min SIMPLE (arrowhead). **b**, **c**. Tissue sections from the brain shown in (b, right panel) were stained with FJC. The degenerating neurons are indicated with the white arrowheads in **b’**, an inset of (**c**). The distribution of the fluorescence between the margins and center of the field is uneven with our stereoscope, which sometimes leads to a darker halo in the middle of the imaged field. **d**. Tissue sections from the brain shown in (b, right panel) were stained with Hoechst33342 to label nuclei. Right, ischemic side; left, contralateral side. **c’** and **d’**, high-magnification images from non-infarct and infarct brain regions, respectively. Shrunken nuclei are indicated with arrows in **d’**

Because most unilateral arterial cerebral infarctions in newborn infants occur in the MCA [2], we tested the ability to produce MP occlusion in the distal MCA (dMCA) of mouse pups. Taking advantage of the transparency of perinatal pup skulls, we identified the tertiary branch of the MCA and glued a micro-magnet onto the skull at the corresponding location. At 20 min post-injection of MP into P3–7 mice (with the dose for permanent occlusion; see Table 1), we removed the micro-magnet and returned the pups to their home cages. After 24 h, we observed that MP aggregation was still present in the dMCA of these mice (Fig. 4a, b).

Intriguingly, we found substantial micro-bleeding in these P3 mouse brains (Fig. 4a), and the infracted brain area was pale in appearance (Fig. 4b), which is a feature characteristic of an ischemic region after the brain is subjected to MCA occlusion [19]. The extent of the pale area accounted for 20% of the hemisphere (Fig. 4b). Compared to brains subjected to 24 h of SIMPLE, brains subjected to 3 days of SIMPLE exhibited a more profound and distinct pale area (Fig. 4b). To examine whether occlusion in the tertiary branch of MCA leads to neuronal degeneration, we stained sections from these brains with FJC. Notably, we found massive neuronal death in the cerebral cortex (Fig. 4c). Using Hoechst33342 staining, we observed shrunken nuclei in the infarcted brain area (Fig. 4d), which is consistent with the results of FJC staining. Thus, we demonstrated that 20 min of SIMPLE with a high dose of MPs produces a permanent focal MP occlusion in the dMCA of perinatal brain and results in massive cell death downstream of the dMCA.

### Reversible occlusion in dMCA by using SIMPLE

**Fig. 5:**
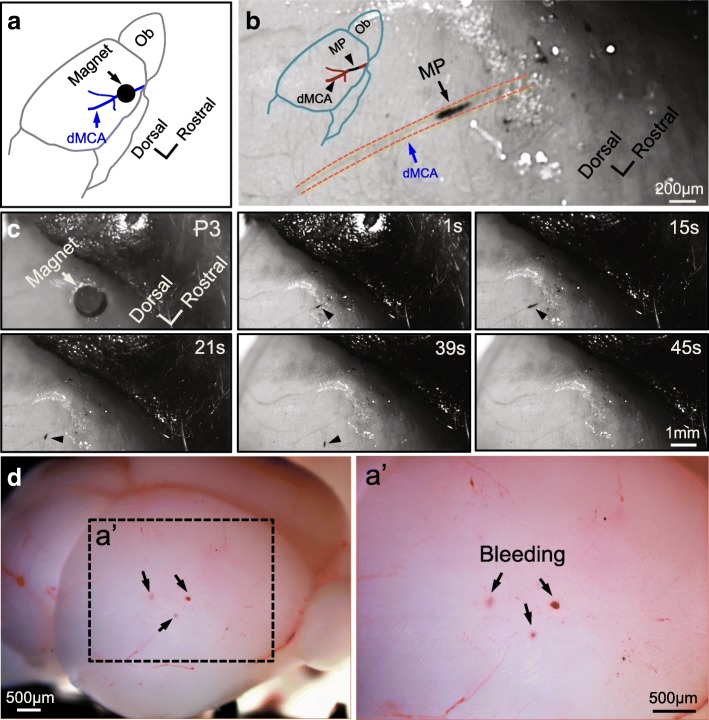
Induction of reversible dMCA occlusion in perinatal mice by SIMPLE. **a**. Illustration of the location of the magnet and dMCA in (**b**) and (**c**). **b**. MPs (black arrowhead) aggregated in the dMCA (outlined with dashed lines) shortly after 20 min SIMPLE with a low dose of MPs (15 μg/g) in a P3 mouse (~ 2 g). **c**. The progress of blood reperfusion at different time points (1 s, 15 s, 21 s, 39 s and 45 s) after the magnet (arrow) was removed after 20 min of occlusion. The location of the MP aggregation is indicated with white arrowheads at the different time points. It has been extremely challenging to do time-lapse imaging of brain vasculature of neonatal pups, because their skulls are very soft and we could not use metal bars to do fixation with stereotaxic. The shift usually occurred during imaging. **d**. The brain dissected from the mouse in **c** at 24 h after it underwent a 20 min reversible occlusion. **a’**, inset from (**d**). Microbleeding was observed in the cerebral cortex

**Fig. 6:**
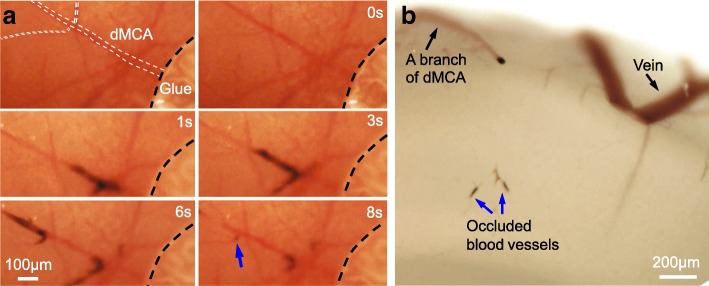
Only a few small blood vessels were occluded by MPs after we removed the magnet from the skull above the dMCA. **a** Time-lapse imaging of occlusion before and 0, 1, 3, 6, and 8 s after removal of the magnet. Dotted line indicates the margin of the glue used to affix the magnet to the skull. **b** Image of brain slice from the pup used for SIMPLE in (**a**) after fixation. dMCA, distal middle cerebral artery. Blue arrows, occluded small blood vessels

To optimize the conditions that produce reversible occlusion in the dMCA of perinatal and neonatal mouse brains, we further decreased the MP injection dose and determined the optimal doses for producing reversible occlusion (as shown in Table 1). Mice of higher body weight need higher doses of MP for reversible occlusion. For example, we injected 10–15 μg/g MPs into P3 mice with 1.8–2.5 g body weight for reversible occlusion. We placed the magnet at the location of the dMCA (Fig. 5a), in which we were able to observe the occlusion status under a dissection microscope. The MP occlusion and reperfusion were recorded in real time. Upon removal of the micro-magnet after a 20 min occlusion (Fig. 5b, c and Additional file 1: Video S1), reperfusion was achieved. We noticed some small vessels (including capillaries) were occluded downstream (< 1 mm away from the occluded region of the MCA) after we removed the magnets from the dMCA (Fig. 6). We sacrificed the mice and harvested the brains 24 h after they were returned to their home cages. We found microbleeding in 40% of the brains (*n* = 5 mice), suggesting that transient ischemia likely led to hemorrhage. We have not detected any bleeding in the contralateral side in any of the mice that we have imaged (*n* = 5 mice). We cut brain slices to check for deeper bleeding and also did not observe bleeding in deeper brain regions (*n* = 5 mice). Taken together, our experiments show that using the SIMPLE technique, we can induce reversible occlusion of the dMCA in mouse brains of P0–7.

## Discussion

Comparisons of neuroanatomy, neurogenesis, gliogenesis, synaptogenesis, and myelination in humans and rodents suggest that P1–5 rodent brains correspond to 23–32 weeks of gestation in humans, thus making this rodent model suitable for studying preterm human brain injury. P7–10 rodent brains correspond to 36–40 weeks of gestation in humans, thus making them suitable for studies of at-term brain injury [7, 20]. There is only a small body of mechanistic studies on stroke in P0–7 mice due to a lack of efficient methods to produce focal ischemic stroke that can be broadly applied to mouse pups, which has long hindered the progress of IPS research. Due to the very small body size/weight of neonatal and perinatal mice, MCAO is not a feasible method to produce focal ischemia in perinatal or neonatal mice younger than P7 [4, 6, 21]. In this study, SIMPLE allowed us to precisely produce occlusion of the dMCA as early as P0 (notably at P0–7), which can facilitate studies of IPS.

Photothrombosis (mediated by Rose Bengal) is frequently used to produce focal ischemic stroke in perinatal and neonatal mice [22, 23]. However, we found both 488-nm and 543-nm lasers excite Rose Bengal and the emission spectrum of Rose Bengal is very wide. In addition, Rose Bengal can pass through blood vessels and enter the brain parenchyma. All regions around blood vessels turn red about 30 min to 1 h after Rose Bengal is administered to blood via the tail vein, which compromises its use with different reporter lines (e.g., Ai14) for live imaging studies. Our approach with magnetic particles in this study can complement photothrombosis in these respects. Compared to conventional methods, SIMPLE is a practical approach that can be conveniently conducted using micro-magnets and MP injection. SIMPLE can produce focal ischemia by reversibly occluding micro-vessels including small arteries and veins without a surgical procedure to the common carotid artery. The size and the location of the temporal superficial vein provide an important accessible vessel for the SIMPLE technique, allowing MP injection into any postnatal mouse.

Our understanding of the neurovascular reorganization after perinatal stroke is incomplete. Although numerous studies about perinatal stroke have been published, there is currently limited knowledge concerning the plasticity of the developing brain after ischemic brain injury, resulting in a lack of efficient treatment strategies for most children after perinatal stroke. Motor deficits are prominent in children with perinatal stroke syndromes, with a prevalence of 30–60% after arterial ischemic stroke [24–26] and 30–50% after cerebral sinovenous thrombosis [27]. It is unclear whether children affected by perinatal stroke suffer morbidity at greater rates than older infants and children [28, 29]. Current treatments for perinatal stroke are usually based on experience and knowledge gleaned from adults. However, the underlying cellular mechanism of ischemic brain injury in the developing brain is significantly different from that of adults. SIMPLE provides an in vivo model for examining the responses of different cellular components of neurovascular units to focal ischemic injury in the developing brain. Combining SIMPLE with advanced cellular imaging and genetic labeling techniques in a mouse model has the potential to elucidate how neurovascular units are reorganized after stroke and advance therapeutic interventions.

## Methods

### Animals

Animal work was conducted in unsexed P0–7 mouse pups with C57BL/6 J background. All animal experiments were performed in accordance with animal protocols approved by the Institutional Animal Care and Use Committee (IACUC) of the University of Texas, Southwestern Medical Center and Westlake University.

### Synthesis of magnetic particles

The magnetic particles were made of ferrite oxide coated with PEG. 180 nm MPs coated with PEG-2000 were used in this study. Magnetic particles were purchased from MicroMod in Germany (Nanomag-D, PEG-2000, 180 nm, stock concentration 10 mg/ml, Cat# 09–54-182, S13014). In the first step of magnetic particle synthesis, the magnetite crystals are covered by dextran. In the next step, the dextran terminals are carboxylated and allowed to react with PEG-2000, leading to the corresponding PEG-2000 ester moieties on the particle surface. Magnetic particles consist of aggregates of 10–20 nm magnetite crystals embedded into a dextran matrix (TEM images are shown in Fig. 1). Thus, the particles can be considered “multi-core” as opposed to “single-core.” In water, these particles behave as spheres stabilized by strong H-bridge interactions between dextran and water. They possess a relatively narrow size distribution in terms of their hydrodynamic diameters (~ 180 nm). This size distribution remains stable after common handling procedures like centrifugation, magnetic separation, sonication, vortex-resuspension, etc. However, the dynamic H-bridges between dextran and water are destroyed and the spherical behavior of these particles is lost after freezing, drying, and exposure to protic solvents, among other things. For example, drying the particles (e.g., in TEM preparation) leads to flake-like structures (Fig. 1).

### Characterization of the magnetic particles

For transmission electron microscopy measurement, MPs (50× dilution) in ultrapure water was transferred on copper grid and dried with tissues wipes, which was then analyzed by a 120 kV JEOL 1400 transmission electron microscope. For dynamic light scattering measurement, MPs (30× dilution) were suspended in ultrapure water and hydrodynamic diameter was obtained with a Malvern Zetasizer Nano ZS particle size analyzer.

### Permanent occlusion induced with magnetic particles

Hypothermia was used to anesthetize P0–7 mouse pups before the surgery was performed. Following a standard anesthesia protocol from the University of Texas Southwestern Medical Center, pups were protected with gloves and immersed in ice. At 4–8 min post-hypothermic anesthesia, SIMPLE was performed according to a previous publication [18] with modifications. A 3 mm incision was made in the skin over the superficial temporal vein. A cylindrical micro-magnet with 1 mm diameter was immobilized on top of the skull with superglue after incision. Due to the transparency of pup skulls, we can identify the location of the dMCA without thinning or removing the skull. The micro-magnet was fixed onto the mouse skull before injecting MPs in 20 μl PBS with an insulin syringe (34-G needle) via the superficial temporal vein. MP occlusion formation strongly relies on two factors: the dose of injected MPs and the duration of SIMPLE. The dosages of MPs for inducing permanent occlusion are listed in Table 1. For experiments in Fig. 2, the pups were sacrificed with an overdose of isoflurane and then their brains were harvested at 10 min to 17 h post-MP injection. For experiments in Fig. 3, we removed the micro-magnet on the skull at 20 min post-injection, sealed the skin with adhesive gel (3 M) and returned the pups back to their home cages. We euthanized the pups 1 d or 7 d later.

### Micro-magnets for SIMPLE

Cylindrical magnets were designed by our laboratory. They were made of neodymium-iron-boron (NdFeB) and produced in China as requested (Zhenlin Co. Ltd., Jiaozuo, Henan Province). The magnets used in this study were cylindrical, 1 mm long and 1 mm in diameter.

### Reversible occlusion induced with magnetic particles

For experiments in Fig. 5, the same experimental paradigm described above was performed with a lower dose of MPs (see Table 1), and the micro-magnet was removed 20 min post-MP injection. Blood reperfusion was recorded in video (SMZ-18, Zyla SCMOS5.5, Nikon) when the micro-magnet was removed.

### Fluoro-jade C staining

Mouse pups that had been subjected to 7 h to 7d of SIMPLE were euthanized by overdose of isoflurane and their brains were fixed with 4% paraformaldehyde (PFA) and dehydrated with sucrose (15% and then 30%). Brain sections (30 μm) were placed on glass slides and air-dried for 30 min. Degenerating neurons were detected with FJC staining as previously described [30]. Brain sections were immersed in a solution with 80% ethanol and 1% NaOH for 5 min, 70% ethanol for 2 min, distilled water for 1 min, 0.06% potassium permanganate (KMnO4) for 15 min and distilled water for 2 min, then incubated in a solution containing 0.001% FJC and 0.1% acetic acid for 15 min at room temperature. The sections were mounted with DPX (Sigma), a non-aqueous, styrene-based mounting medium.

### Fluorescent immunostaining

Fixed mouse brains were sectioned with a cryostat (model CM3050S, Leica). Brain sections were stained as we described previously [31]. Briefly, sections were permeabilized with 0.25% Triton X-100 followed by a blocking solution of 5% bovine serum albumin and 3% normal goat serum with 0.125% Triton X-100 for 2 h. Primary antibodies against Tbr1 (1:200, 1:150, rabbit, polyclonal, Cat^#^ ab31940, Abcam) or NeuN (1:300, rabbit, monoclonal, Cat. No. MAB377, EMD Millipore) were incubated with brain sections for 24–48 h at 4 °C. In conjunction with staining with Hoechst 33,342 (0.5 μg/ml), brain sections were incubated with secondary antibodies conjugated with Alexa488 or Alexa543 (1:600 dilution, Thermo Fisher Scientific) for 2 h at room temperature (22–25 °C). Sections were mounted with the anti-fade mounting medium Fluoro-Gel (EMS), and images were acquired with a Zeiss LSM710NLO confocal microscope.

## Additional file

Additional file 1: Real-time imaging of the MP occlusion and reperfusion. Upon removal of the micro-magnet after a 20 min occlusion, reperfusion was achieved. (MOV 682 kb)

## Abbreviations

dMCA: Distal middle cerebral artery
IPS: Ischemic perinatal stroke
MCA: The middle cerebral artery
MCAO: Middle cerebral artery occlusion
MP: Magnetic nanoparticles
SIMPLE: Stroke Induced with Magnetic Particles
